# Elevated serum fibroblast growth factor 21 levels correlate with immune recovery but not mitochondrial dysfunction in HIV infection

**DOI:** 10.1186/1742-6405-10-27

**Published:** 2013-11-19

**Authors:** Brendan AI Payne, David Ashley Price, Patrick F Chinnery

**Affiliations:** 1 Institute of Genetic Medicine, Newcastle University, Central Parkway, Newcastle-upon-Tyne NE1 3BZ, UK; 2 Department of Infection and Tropical Medicine, Royal Victoria Infirmary, Newcastle-upon-Tyne NE1 4LP, UK

**Keywords:** HIV, Anti-retroviral therapy, Highly active, Fibroblast growth factor 21, Mitochondria

## Abstract

**Background:**

Anti-retroviral treated HIV-infected patients are at risk of mitochondrial toxicity, but non-invasive markers are lacking. Serum FGF-21 (fibroblast growth factor 21) levels correlate strongly with muscle biopsy findings in inherited mitochondrial disorders. We therefore aimed to determine whether serum FGF-21 levels correlate with muscle mitochondrial dysfunction in HIV-infected patients.

**Findings:**

We performed a cross-sectional study of anti-retroviral treated HIV-infected subjects (aged 29 – 71 years, n = 32). Serum FGF-21 levels were determined by quantitative ELISA. Cellular mitochondrial dysfunction was assessed by COX (cytochrome *c* oxidase) histochemistry of lower limb skeletal muscle biopsy. Serum FGF-21 levels were elevated in 66% of subjects. Levels correlated significantly with current CD4 lymphocyte count (p = 0.042) and with total CD4 count gain since initiation of anti-retroviral therapy (p = 0.016), but not with the nature or duration of past or current anti-retroviral treatment. There was no correlation between serum FGF-21 levels and severity of the muscle mitochondrial (COX) defect.

**Conclusions:**

Serum FGF-21 levels are a poor predictor of muscle mitochondrial dysfunction in contemporary anti-retroviral treated patients. Serum FGF-21 levels are nevertheless commonly elevated, in association with the degree of immune recovery, suggesting a non-mitochondrial metabolic disturbance with potential implications for future comorbidity.

## Findings

Mitochondrial dysfunction is a well-described complication of anti-retroviral therapy [1-7]. It is most strongly associated with several of the older nucleoside analogue reverse transcriptase inhibitors (NRTIs): zidovudine (AZT), stavudine (d4T), didanosine (ddI), and zalcitabine (ddC). Although these drugs are no longer in common usage in industrialised countries, there are nevertheless large numbers of patients who have had extensive prior exposure to these drugs, and some remain in common usage in developing countries. We have recently demonstrated that patients with previous exposure to these NRTIs may have persistent cellular mitochondrial COX (cytochrome *c* oxidase) defects in skeletal muscle, consequent on an NRTI-induced accumulation of somatic (acquired) mitochondrial DNA (mtDNA) mutations [8].

Non-invasive measures of mitochondrial damage would be very valuable in the HIV clinic, both for the diagnosis of anti-retroviral associated mitochondrial dysfunction and the serial monitoring of such patients. The determination of mtDNA content in peripheral blood mononuclear cells (PBMCs) has previously been proposed as such a measure [5,9-11]. This consideration arises from the fact that the mitochondrially-toxic NRTIs (as listed above) cause a reduction in cellular mtDNA content (depletion) during therapy [2,3,6,10,12-16]. However, modern N(t)RTIs such as tenofovir (TDF) and abacavir (ABC) do not cause mtDNA depletion [17], and as a result, mtDNA levels return to normal with a switch away from a mitochondrially-toxic NRTI. Thus, measuring mtDNA levels is not a useful measure of on-going mitochondrial dysfunction due to an NRTI exposure in the distant past.

In contrast, FGF-21 (fibroblast growth factor 21) has recently been proposed as a valuable serum measure in inherited mitochondrial disease [18]. In these patients, serum FGF-21 levels showed a very strong correlation with mitochondrial dysfunction on skeletal muscle biopsy, as determined by the percentage of cells expressing a COX defect [18]. FGF-21 is thought to regulate mitochondrial activity and enhance oxidative capacity, mediated via PGC-1α (peroxisome proliferator-activated receptor gamma co-activator 1-alpha) expression [19]. To date, one study has assessed serum FGF-21 in HIV infection, and demonstrated elevated levels [20]. Given the recently described association between serum FGF-21 elevation and muscle COX defects in inherited mitochondrial disorders [18], and the recent observation of significant COX defects in long-term anti-retroviral treated HIV-infected patients [8], we speculated that muscle mitochondrial dysfunction might also drive the FGF-21 elevation in anti-retroviral treated HIV infection.

### Patient characteristics

All subjects gave informed written consent for participation, and the study was approved by local research ethics committee (Newcastle and North Tyneside Research Ethics Committee). We performed a cross-sectional study of adult HIV-1 infected patients, receiving ambulatory care at one of two specialist clinics in Newcastle-upon-Tyne, UK. Patients with current active hepatitis B or C co-infection, known inherited or non-HIV-associated neuromuscular disease, and diabetes mellitus were excluded. No subjects were clinically obese (BMI >30). 32 HIV-infected subjects participated, of whom 81% were male. 84% were of white Caucasian ethnicity and the remainder black African. Mean age was 48.7 years, with age range of 29–71 years. Mean duration of diagnosed HIV infection was 10.8 years. Mean current CD4 lymphocyte count was 663 cells/μl, and 61% of subjects had nadir CD4 count of <200 cells/μl. All subjects were currently receiving combination anti-retroviral therapy, with a mean duration of treatment of 9.2 years. 97% of patients had fully suppressed HIV plasma viral load (<50 HIV-1 RNA copies/ml). 81% of subjects were receiving a non-nucleoside reverse transcriptase inhibitor (NNRTI) and 22% a ritonavir-boosted protease inhibitor (PI). Regarding past (lifetime) NRTI treatment experience, 72% of patients had a history of AZT exposure, and 25% had a history of prior d-drug (dideoxynucleoside analogue) exposure. Characteristics of individual subjects are shown in Table 1.

**Table 1:** Patient characteristics

**Age (y)**	**Gender**	**Ethnicity**	**Duration of diagnosed HIV (mo)**	**ART duration (mo)**	**Nadir CD4 count (cells/uL)**	**Current CD4 count (cells/uL)**	**LDS**	**ART (current)**	**ART (lifetime)**	**COX defect (%)**	**Serum FGF-21 (pg/mL)**
71	M	WB	130	130	UK	530	Y	TDF FTC EFV	ddi AZT 3TC EFV TDF FTC	3.0%	232
48	F	BA	100	99	10	487	N	TDF FTC NVP	AZT 3TC EFVTDF FTC NVP	0.1%	>1920
34	F	WB	88	86	218	1121	Y	ABC 3TC NVP	AZT 3TC NVP ABC	0.0%	560
55	F	BA	64	27	112	426	N	TDF FTC AZT DRV/r	TDF FTC LPV/r AZT DRV/r	0.8%	164
43	M	BA	87	87	152	306	N	TDF FTC EFV	AZT 3TC EFV TDF FTC	0.8%	20
42	M	WB	185	147	150	636	N	AZT 3TC EFV	AZT 3TC NVP	0.0%	342
63	M	WB	97	97	169	870	N	ABC 3TC EFV	AZT 3TC EFV ABC	0.0%	204
29	M	WB	84	32	197	401	N	TDF FTC EFV	TDF FTC EFV	0.0%	809
63	M	WB	238	221	NA	438	N	ABC 3TC NVP	AZT ddl d4T 3TC ddC IDV NVP ABC	2.2%	440
62	M	WB	63	62	56	190	N	TDF FTC NVP	TOP FTC NVP	0.2%	156
52	M	WB	225	225	120	728	*Y*	TDF FTC ATV/r	AZT ddC ddl 3TC d4T SQV NVP IDV NFV ABC TDF LPV/r FTC ATV/r	1.3%	328
36	M	WB	139	138	197	627	N	AZT 3TC EFV	AZT 3TC EFV TDF FTC	0.3%	214
51	M	WB	190	183	10	747	N	ABC TDF NVP	AZT ddl d4T 3TC RTV NVP IDV ddC ABC ATV/r TDF	4.9%	252
33	F	WB	96	95	83	1289	N	TDF FTC EFV	AZT 3TC EFV TDF FTC	0.0%	177
48	M	WB	102	100	259	1329	Y	AZT 3TC EFV	AZT 3TC EFV	0.4%	409
51	M	WB	*145*	144	151	421	N	AZT 3TC NVP	AZT 3TC NVP	1.4%	254
66	M	WB	71	26	287	455	N	TDF FTC EFV	TDF FTC EFV	11.2%	470
46	M	WB	158	157	250	1452	N	TDF FTC EFV	AZT 3TC IDV EFV ABC TDF FTC	1.4%	1218
61	M	WB	116	113	NA	498	Y	TDF FTC NVP	AZT 3TC EFV NVP TDF FTC	2.4%	182-
30	M	WB	88	23	283	661	N	TDF FTC DRV/r	TDF FTC EFV DRV/r	0.1%	207
62	M	WB	284	202	NA	422	N	ABC NVP LPV/r	SQV AZT ddC 3TC d4T ddl IDV ABC NVP NFV LPV/r	0.8%	92
45		WB	159	158	176	660	N	TDF FTC NVP	AZT 3TC IDV NVP TDF FTC	0.0%	60
54	M	WB	79	38	244	638	N	TDF FTC DRV/r	TDF FTC EFV DRV/r	3.4%	178
52	M	WB	166	164	0	662	Y	TDF FTC NVP	AZT d4T IDV NFV SQV 3TC NVP ddl TDF FTC	2.8%	525
51	M	WB	243	171	327	539	Y	TDF FTC EFV	AZT ddl RTV NFV TDF FTC EFV	1.5%	231
35	F	BA	62	25	380	638	N	TDF FTC EFV	TOP FTC EFV	0.0%	82
53	M	WB	NA	48	301	804	N	TDF FTC EFV	TDF FTC EFV	NA	490
36	M	WB	130	130	18	898	N	TDF FTC ATV/r	AZT 3TC EFV TDF FTC ATV/r	0.0%	530
48	M	WB	53	14	332	443	N	TDF FTC EFV	TDF FTC EFV	0.0%	56
52	F	BA	83	81	17	485	Y	TDF FTC EFV	AZT 3TC EFV TDF FTC	0.7%	224
38	M	WB	129	128	4	761	Y	TDF FTC EFV	AZT 3TC EFV TDF FTC	0.0%	935
47	M	WB	183	164	305	668	Y	ABC RAL ATV/r	d4T 3TC NVP ddl IDV ABC ATV/r RAL	9.8%	114

Summary characteristics of individual HIV-infected subjects. (WB, white British; BA, black African; ART, anti-retroviral therapy; LDS, lipodystrophy syndrome; AZT, zidovudine; d4T, stavudine; ddI, didanosine; ddC, zalcitabine; 3TC, lamivudine; ABC, abacavir; TDF, tenofovir; FTC, emtricitabine; EFV, efavirenz; NVP, nevirapine; ATV, atazanavir; DRV, darunavir; LPV, lopinavir; SQV, saquinavir; NFV, nelfinavir; IDV, indinavir; RTV, ritonavir at therapeutic dose; /r, ritonavir at pharmacokinetic boosting dose; RAL, raltegravir; COX, cytochrome c oxidase; FGF-21, fibroblast growth factor 21; NA, not available).

### FGF-21 determination

**Figure 1:**
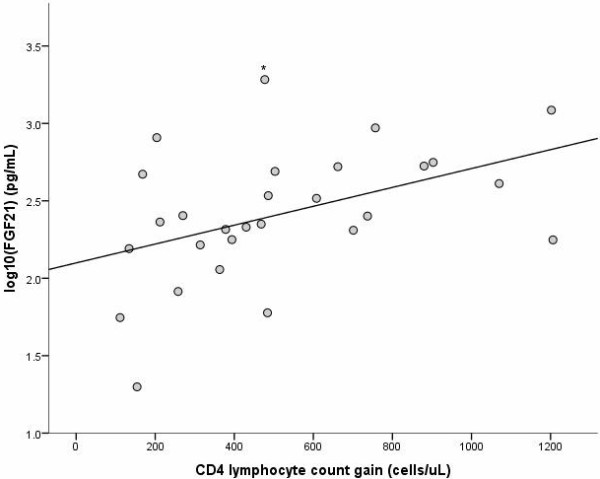
**Correlation of serum FGF-21 levels with immune reconstitution.** Correlation of log_10_ serum FGF-21 (fibroblast growth factor 21) levels in HIV-infected subjects and CD4 lymphocyte count gain on treatment (current minus nadir) (r = 0.45, p = 0.016). (* Serum FGF-21 >1920 pg/ml, the upper limit of quantitation of the assay).

**Figure 2:**
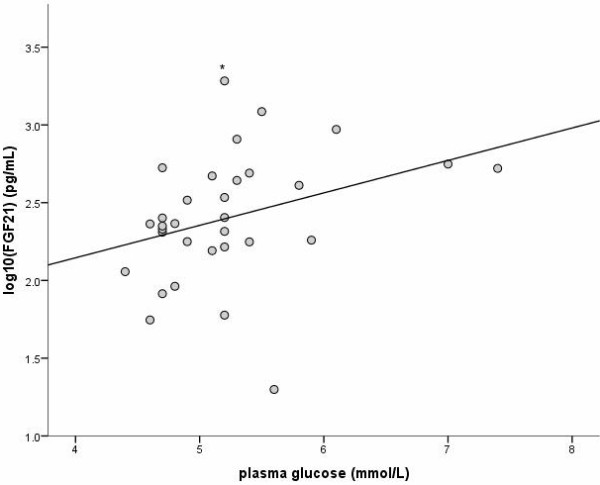
**Correlation of serum FGF-21 levels with plasma glucose.** Correlation of log_10_ serum FGF-21 (fibroblast growth factor 21) levels in HIV-infected subjects and random plasma glucose concentration (r = 0.34, p = 0.06). (* Serum FGF-21 >1920 pg/ml, the upper limit of quantitation of the assay).

Serum FGF-21 levels were determined by quantitative ELISA (BioVendor, Brno, Czech Republic), performed in triplicate, and normalised by log_10_ transformation. A serum FGF-21 level of <200 pg/ml was considered as normal in keeping with recent data [18]. Statistical analyses were performed in SPSS 19, using student’s t-test to compare binary variables and Pearson’s correlation coefficient (r) to examine the relationship between log_10_ serum FGF-21 levels and continuous variables. Twenty-one of 32 subjects (66%) had serum FGF-21 levels greater than the normal range, with four being very elevated (>800 pg/ml). On univariate analysis, serum FGF-21 levels were positively correlated with current CD4 lymphocyte count (r = 0.36, p = 0.042), but more strongly correlated with total CD4 cell count gain since initiation of anti-retroviral therapy (current minus nadir) (r = 0.45, p = 0.016) (Figure 1). In addition, plasma glucose levels correlated with serum FGF-21 levels, although this did not quite reach statistical significance (r = 0.34, p = 0.06, Figure 2), whereas as serum lipids and liver function did not. No other demographic or treatment variables were significantly associated with serum FGF-21 levels, including the nature of current or prior anti-retroviral therapy (Table 2). FGF-21 levels did not differ significantly between patients with or without clinical lipodystrophy syndrome. Only CD4 lymphocyte count gain was independently associated with serum FGF-21 levels on multivariate linear regression analysis (p = 0.016).

**Table 2:** Associations of serum FGF-21 levels

**(a)**			
**Variable**	**(n)**	**Log**_**10**_**Serum FGF-21, mean (SD)**	**p value**
Gender	Male (26)	2.39 (0.40)	
	Female (6)	2.46 (0.48)	0.71
Ethnicity	Caucasian (27)	2.44 (0.33)	
	Black African (5)	2.21 (0.72)	0.27
Current ART	PI (7)	2.29 (0.26)	
	No PI (25)	2.43 (0.44)	0.43
Lifetime ART	d-drugs (8)	2.38 (0.26)	
	No d-drugs (24)	2.41 (0.45)	0.86
	AZT (23)	2.44 (0.42)	
	No AZT (9)	2.30 (0.39)	0.41
Lipodystrophy	Yes (10)	2.50 (0.27)	
	No (22)	2.36 (0.46)	0.39
Lipid-lowering therapy	Yes (8)	2.32 (0.52)	
	No (24)	2.43 (0.38)	0.53
**(b)**			
**Variable**		**Correlation coefficient (r)**	**p value**
Age		−0.07	0.69
Duration of diagnosed HIV infection		−0.08	0.67
Duration of lifetime ART	Total	0.12	0.50
	d-drug	0.01	0.97
	AZT	0.13	0.47
CD4 lymphocyte count	Nadir	−0.29	0.14
	Current	**0.36**	**0.042**
CD4 count gain	(Current minus nadir)	**0.45**	**0.016**
Serum ALT		0.27	0.14
Plasma glucose		**0.34**	**0.06**
Serum lipids	Total cholesterol	−0.03	0.87
	HDL cholesterol	0.02	0.90
	Non-HDL cholesterol	−0.06	0.75
Mitochondrial histochemistry	COX defect (log_10_)	−0.02	0.91

(a) binary variables; (b) continuous variables. (PI, protease inhibitor; AZT, zidovudine; d-drug, dideoxynucleoside analogue; ART, anti-retroviral therapy; ALT, alanine transaminase; HDL, high density lipoprotein).

Bold type indicates statistically significant associations.

### Skeletal muscle mitochondrial histochemistry

**Figure 3:**
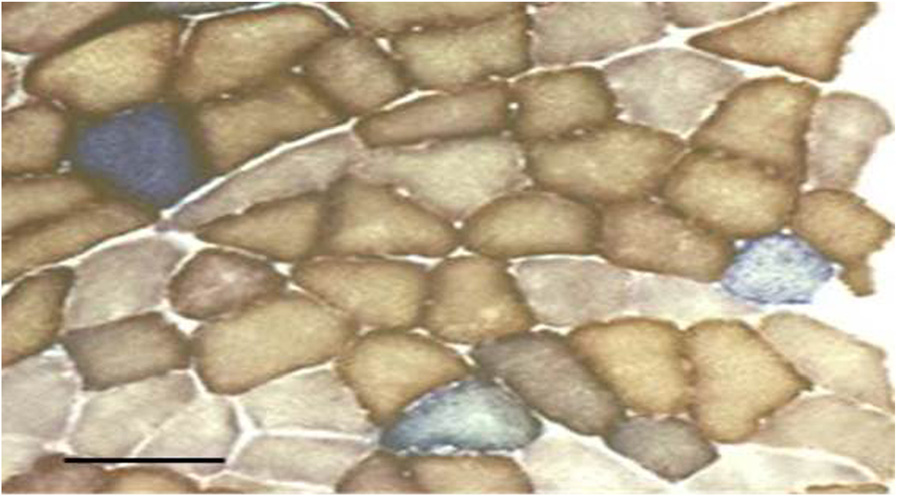
**COX histochemistry.** Example of mitochondrial COX/SDH (cytochrome c oxidase/succinate dehydrogenase) histochemistry on lower limb skeletal muscle biopsy of an anti-retroviral treated HIV-infected patient. Normal (COX positive) fibres stain brown, whereas COX deficient fibres counterstain blue due to preserved SDH activity.

**Figure 4:**
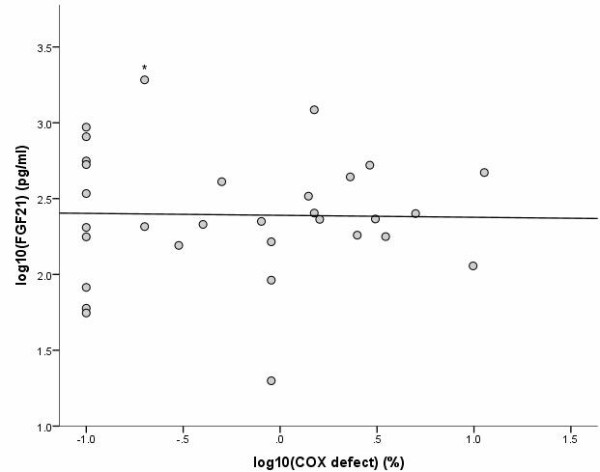
**Correlation of serum FGF-21 levels and mitochondrial defects.** Correlation of log_10_ serum FGF-21 (fibroblast growth factor 21) levels in HIV-infected subjects and percentage COX (cytochrome *c* oxidase) defect on lower limb skeletal muscle biopsy (r = -0.02, p = 0.9). (* Serum FGF-21 >1920 pg/ml, the upper limit of quantitation of the assay).

COX histochemistry was performed on cryo-sections obtained from lower limb skeletal muscle biopsies on 31 of the 32 subjects (biopsy data for one subject was not analysable). Results of 22 of these biopsies have been reported in our previous work [8], whereas the remaining 9 have not. COX contains respiratory chain subunits encoded by the mitochondrial genome, and fibres stain brown (positive) in the presence of intact respiratory chain activity (Figure 3). Proportional COX defect was determined by counting ≥500 fibres per biopsy, and normalised by log_10_ transformation. There was no correlation between serum FGF-21 levels and percentage COX defects on biopsy (r = −0.02, p = 0.9, Figure 4).

### Discussion

We have shown that serum FGF-21 levels are frequently elevated in contemporary anti-retroviral treated HIV-infected patients, but do not correlate with the severity of muscle mitochondrial (COX) defect. In contrast, a previous study has shown a very strong correlation between these parameters in patients with inherited mitochondrial disorders [18]. Ours is the first study to attempt to link serum FGF-21 levels with biopsy-proven mitochondrial defects in HIV-infected patients. What is the reason for this apparent discrepancy in findings? Firstly, the prior study demonstrating serum FGF-21 elevation in mitochondrial disease included a large number of patients with childhood-onset disease. Such patients typically have very severe muscle COX defects (affecting up to ~60% of fibres). In contrast, patients with late-onset inherited mitochondrial disorders typically have more modest COX defects, comparable with those seen in our HIV-infected patients (up to ~10% of fibres). The fact that some patients in our study with a biopsy COX defect of >5% of fibres had relatively normal FGF-21 levels suggests that this serum measure is not particularly sensitive for mild to moderate muscle mitochondrial defects. Secondly, the markedly abnormal serum FGF-21 levels seen in some patients with no significant COX defect suggest a non-mitochondrial origin, as has been observed in other metabolic disorders [21-23]. In the only previous study of FGF-21 levels in HIV infection, the authors found associations of FGF-21 levels with obesity, glycaemia, dyslipidaemia and liver dysfunction, in line with literature from HIV-uninfected patients [24]. In our study, we specifically excluded diabetic and obese subjects (as we wished to maximise the likelihood of detecting any association with NRTI-induced mitochondrial dysfunction). Interestingly however, the strongest predictor of serum FGF-21 levels seen in our study was a novel association with total CD4 lymphocyte count gain. This is an intriguing finding. It is plausible that patients who have low nadir CD4 lymphocyte counts may experience more profound metabolic changes as they undergo immune reconstitution on anti-retroviral therapy, switching from a catabolic state to an excessively anabolic state associated with a ‘return to health’. This association with CD4 count gain should be further explored by longitudinal study.

In conclusion, serum FGF-21 levels do not appear to be a sensitive or specific marker of muscle mitochondrial dysfunction in contemporary anti-retroviral treated patients. Nevertheless they are commonly elevated in association with immune recovery. As serum FGF-21 levels in the HIV-uninfected population are elevated in conditions associated with increased cardiovascular risk, it is very plausible that serum FGF-21 elevation in anti-retroviral treated HIV infection may also be a marker of an adverse metabolic risk in this patient group. Given the known increase in cardiovascular disease in anti-retroviral treated patients [25], the prognostic significance of our findings merits further research.

## Competing interests

All authors confirm that they have no relevant competing interests.

## Authors’ contributions

BP conceived the study, performed the assays and drafted the manuscript. DAP conceived and helped coordinate the study. PFC conceived the study and drafted the manuscript. All authors read and approved the final manuscript.
